# Supplementary material for: An exploratory study on becoming a traditional spiritual healer among Baganda in Central Uganda
Source: PLOS Glob Public Health. 2024 Apr 25;4(4):e0002581. doi: 10.1371/journal.pgph.0002581 (PMC11045116; doi:10.1371/journal.pgph.0002581)
Supplement: S1 Text — (DOCX) [file pgph.0002581.s001.docx]

# **Contextual Definitions (Legends)**

In the context of this study, the definitions given here below are based mainly on the field findings.

**Ancestors** refer to persons that have passed on to other dimensions of existence, for whom people are descendants. They include relatives such deceased parents, grandparents, great-grandparents, aunts and uncles, animals, birds and stones

**Ancestral spirits** refer to guardian spirits that possess people and talk through them as medium. They also guide through dreams and these include *Mizimu*, *Mayembe* and *Misambwa.*

**Animals** in the context of this study, refer to low-conscious living things in form of animals, reptiles, insects, birds, etc. empowered with spiritual powers which are used in health care by the traditional spiritual healers

**Apprentice** is formal hands-on-training, a traditional spiritual healer trainee undergoes, while staying and working closely with a senior specialised spiritualist, to learn how to interact with the ancestral spirits, to know and use ancestral spirits to manage and heal cultural and spiritual health issues.

**Baganda** (singular *Muganda*) are an ethnic group of Bantu speaking people of Buganda kingdom in Central Uganda, with a recognised hereditary leader known as the Kabaka. They consist of clans, sub-clans and families linked by social and blood ties, with a common culture, and speak *Luganda*. as their language.

**Beliefs** refer to what Baganda traditional spiritual healers thought and trusted as true or correct with or without guiding reason to one’s decision, action or behaviour.

**Diagnostic tools** refer to a set of objects, upon which rituals are performed, used by traditional spiritual healers to understand the nature of problem, its root cause and the ways to manage the problem. For example, the use of coffee beans and cowrie shells.

**Disease** refers to affected body parts that manifest signs, symptoms such as pain and fever.

**Divination** refers to cultural process to foretell and give advice based on retrospective and prospective foresight by traditional spiritual healers

**Dreams** are images, ideas, feelings or thoughts that flow while one soul travels to the spiritual realms when the body is sleeping.

**Health** refers to the resultant situation of the harmonized and integrated human life with other low-conscious living things, the environment, and the sociocultural, spiritual and metaphysical factors in nature.

**Health care** refers to socially acceptable efforts made to maintain or restore the physical, mental, emotional, psychological, financial, spiritual and social well-being of individuals, families or community.

**Illness** refers to the subjective ways practitioners understand the manifestations of the affected soul and spirit parts, the root cause, and management.

**Initiation** is a rite of passage spiritualists go through that involve spirit-specified rituals and tests to confirm his/her connections with the ancestral spirits.

**Intuition** is the ability for one to understand through spontaneous instinctive feelings of smell, touch, hearing, knowing, seeing without necessarily using facts, evidence nor conscious reasoning.

***Lubaale*** refers to individual or collective ancestral spirits.

***Mayembe*** (*Jjembe* singular) refer to ancestral spiritual entities headed by *Jjembe* Lubowa. They assist on various roles and responsibilities utilized as worker spirits

**Mediums** refers to animals or human beings utilized by ancestral spirits and act as intermediary channels of communication between ancestral spirits and human beings.

***Misambwa* (***Musambwa-singular***)** are most superior categorized ancestral spirits with persistent impressions in the communities.

***Muzimu (****Mizimu-Plural****)*** is the collective consciousness of that person who passed on to the spiritual realm mainly characterised by the soul.

**Offering** refers to anything intentionally given out for ritualistic purpose towards ancestral spirits and other supernatural forces.

**Plants** in the context of this study refer to vegetation empowered with spiritual powers used in health management by the traditional spiritual healers

**Regalia** refers to distinctive traditional ritualistic clothing and ornament items of cultural strength and significance that symbolize rooted relationship and communication.

**Rituals** are recognised cultural practices rooted in tribal spirituality.

**Ritual healing** is traditional medicine practice rooted in cultural spirituality that may involve a wide range of formalities and materials used to manage the physical, mental, emotional, financial, spiritual and psychological difficulties.

**Sacred places** are dwelling places for spiritual beings, imbued with spiritual, supernatural and mystical powers and abilities. Examples include places such as natural forests, rivers, mountains, shrines, and fire-places.

**Sacrifice** refers to intentional giving of animals and/or birds for ritualistic purpose towards ancestral spirits, supernatural forces and divine beings.

**Spirituality** refers to ancestral, supernatural, inborn, involuntary and practical reality that guides people and society based on the values, meanings and relationships to the sacred by which people live.

**Symbol** is a representation of spiritual information, connectivity and power contained in a material and associated ancestral spirits. For example, ornament items, ritual cloths, and colors

**Baganda traditional spiritual healers (*Balubaale*)** are native health practitioners, able to communicate with ancestral spirits as medium, for health management of individuals, families and communities using spiritual powers embedded in plants, animals and sacred places.

**Voices** refer to verbal sound “heard” through ears
